# Unicentric Castleman disease with paraneoplastic pemphigus in a young woman: a case report

**DOI:** 10.3389/fmed.2025.1749601

**Published:** 2026-01-16

**Authors:** Guan-ting Lv, Yi-hang Zhao, Jia-li Zhao, Zhi-yi Zheng, Zi-jun Liu

**Affiliations:** Department of General Surgery, Nanjing First Hospital, Nanjing Medical University, Nanjing, Jiangsu, China

**Keywords:** case report, paraneoplastic pemphigus, prognosis, surgery, unicentric Castleman disease

## Abstract

**Introduction:**

The case reports a rare case of a patient diagnosed with unicentric Castleman disease (UCD) with paraneoplastic pemphigus (PNP). According to the literature, surgery is considered the first-line therapy for such patient. Therefore, we collected and analyzed preoperative and postoperative data to evaluate the surgical outcome and prognosis.

**Case presentation:**

A 28-year-old female patient had manifested erosions on oral mucosa and fingers for several months. Contrast-enhanced CT imaging revealed an abdominal mass. Histopathology of the lesions revealed lichenoid interface dermatitis. Direct immunofluorescence examination of the skin revealed a reticular deposition of IgG and C3 in the intercellular spaces of the epidermis. According to the latest diagnostic criteria, she was diagnosed with PNP. She underwent surgery at our hospital, during which the mass was completely resected. Postoperative pathology confirmed the mass as UCD, specifically of the hyaline vascular type. Following the surgery, the patient was concurrently treated with a tapering regimen of oral glucocorticoids. After 2 years of follow-up, there was no recurrence of the oral or hand erosions, and a repeat abdominal CT scan showed no evidence of mass recurrence.

**Conclusion:**

Complete resection is the first-line therapy for UCD with PNP. Early and correct diagnosis is crucial for patient prognosis.

## Background

Castleman disease (CD) is a heterogeneous disorder primarily characterized by lymph node enlargement, first described by Castleman in 1954 (1). Epidemiological data indicate that the incidence rate of CD is approximately 22–25 per million person-years (2). CD can involve lymph nodes throughout the body. Based on the distribution of affected lymph nodes, it is classified into unicentric Castleman disease (UCD) and multicentric Castleman disease (MCD) (3, 4). The clinical manifestations of these two subtypes differ significantly. Patients with UCD typically present with an enlarged single lymph node or a solitary group of lymph nodes and are usually asymptomatic (3). After complete resection, the prognosis of patients is usually favorable and the recurrence rate is low. The 5-year survival rate for UCD patients after complete resection exceeds 90% (5–7).

Paraneoplastic pemphigus (PNP) is a rare autoimmune disorder associated with underlying neoplasm. PNP is frequently associated with lymphoproliferative neoplasms. The majority of the neoplasms reported in PNP include non-Hodgkin’s lymphoma, chronic lymphocytic leukemia, CD, and thymoma (8). PNP is clinically characterized by painful stomatitis and polymorphous cutaneous lesions. Patients with PNP can present with oral lesions, other mucosal lesions, skin lesions and pulmonary manifestations (9). Oral lesions are the earliest manifestation and are present in almost all PNP patients (10–12). CD is the most common neoplasm in PNP patients with oral manifestations (10). Oral manifestations can range from blisters, erosions to diffuse painful stomatitis involving the lips, tongue, cheeks and gingivae or the entire oral cavity (13, 14). Painful ulcers on the tongue and vermillion border on the lips are also obvious clinical features (14). Moreover, nasal mucosa, pharynx, larynx or esophagus can be involved, causing odynophagia and dysphagia, seriously influence the quality of life, nutrition status of patients (8, 15, 16). Although repeated course of systematic corticosteroids may relieve the oral pain, lesions may recur and worsen (10, 11).

The progression of untreated PNP may lead to bronchiolitis obliterans (BO), which is life threatening and associated with poor prognosis (12, 17). Since immunosuppressive treatment alone is ineffective without treating the underlying neoplasm, complete surgical removal of the tumor should be performed as soon as possible, ideally before the onset of BO (18). PNP can progress even after the original malignancy is removed (9). However, PNP with CD shows better outcomes after removing the mass in surgery. Once the mass is removed, skin and mucosa erosions typically subside gradually (19).

## Case presentation

A 28-year-old female patient presented with the erosions of multiple fingers and extensive painful oral erosions for several months. Treatment with oral hydroxychloroquine and prednisone, along with topical triamcinolone acetonide, yielded no significant improvement, prompting her referral to our institution for further evaluation. The patient denied fever, dyspnea abdominal distension, diarrhea, dyspnea or related symptoms of respiratory system. However, the worsening oral pain impaired the daily eating and leads to a weight loss of 7.5 kg.

**FIGURE 1:**
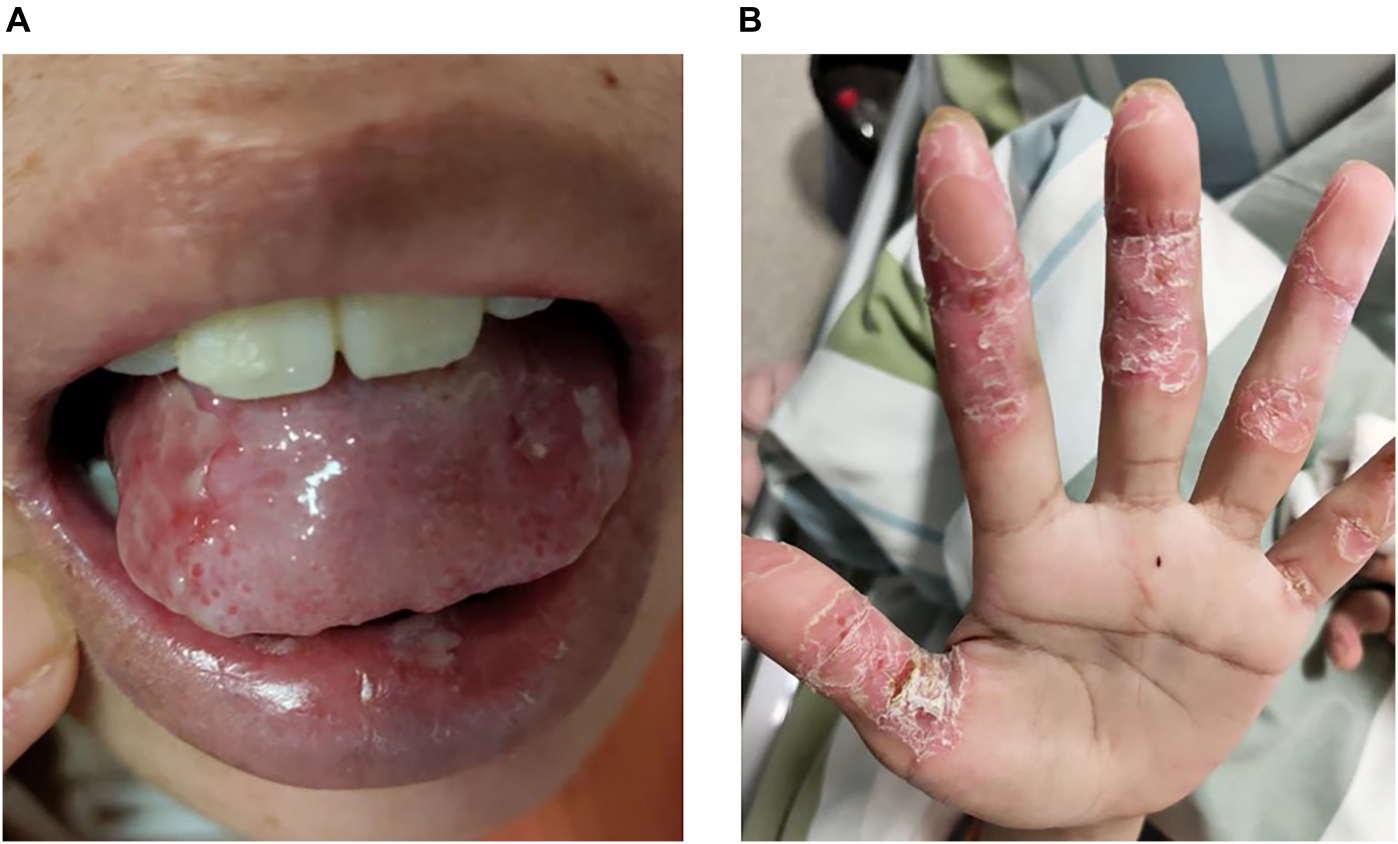
Erosions on the **(A)** tongue and **(B)** fingers.

**FIGURE 2:**
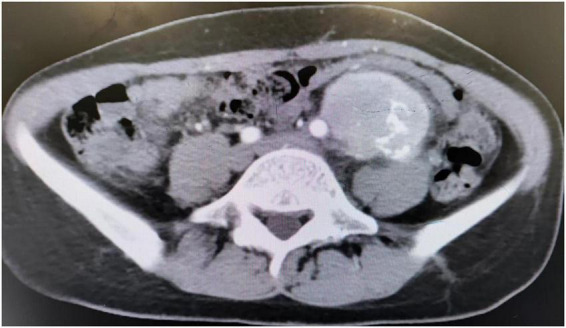
CT scan showed an abdominal mass.

Multiple erosions were observed on the oral mucosa and tongue (Figure 1A), as well as on the fingers of both hands (Figure 1B). The physical examination of abdomen revealed no abnormal results. The abdomen was flat with no scars. There was no tenderness or rebound tenderness throughout the abdomen. A firm mass was palpable in the left lower quadrant, with a clear boundary from the surrounding tissues. A biopsy of a finger erosion was consistent with lichen planus. A contrast-enhanced abdominal CT scan revealed a solitary mass in the left lower abdomen measuring 5.4 cm × 5.1 cm × 9.2 cm, with a clear boundary from the surrounding tissues (Figure 2). The patient’s biochemical profile, full blood count, serum antibodies (IgA, IgG, and C3) and coagulation profile were normal with no significant abnormalities detected. The result of the human immunodeficiency virus (HIV) screening test was also negative. One of the tumor markers was abnormal, the squamous cell carcinoma-associated antigen level was significantly elevated at >70 ng/ml (normal range: <1.5 ng/ml). Direct immunofluorescence examination of the skin revealed a reticular deposition of IgG and C3 in the intercellular spaces of the epidermis. Histopathology of the lesions revealed lichenoid interface dermatitis. According to the diagnostic criteria established by the European Academy of Dermatology and Venereology in 2023, she was diagnosed with PNP (12).

**FIGURE 3:**
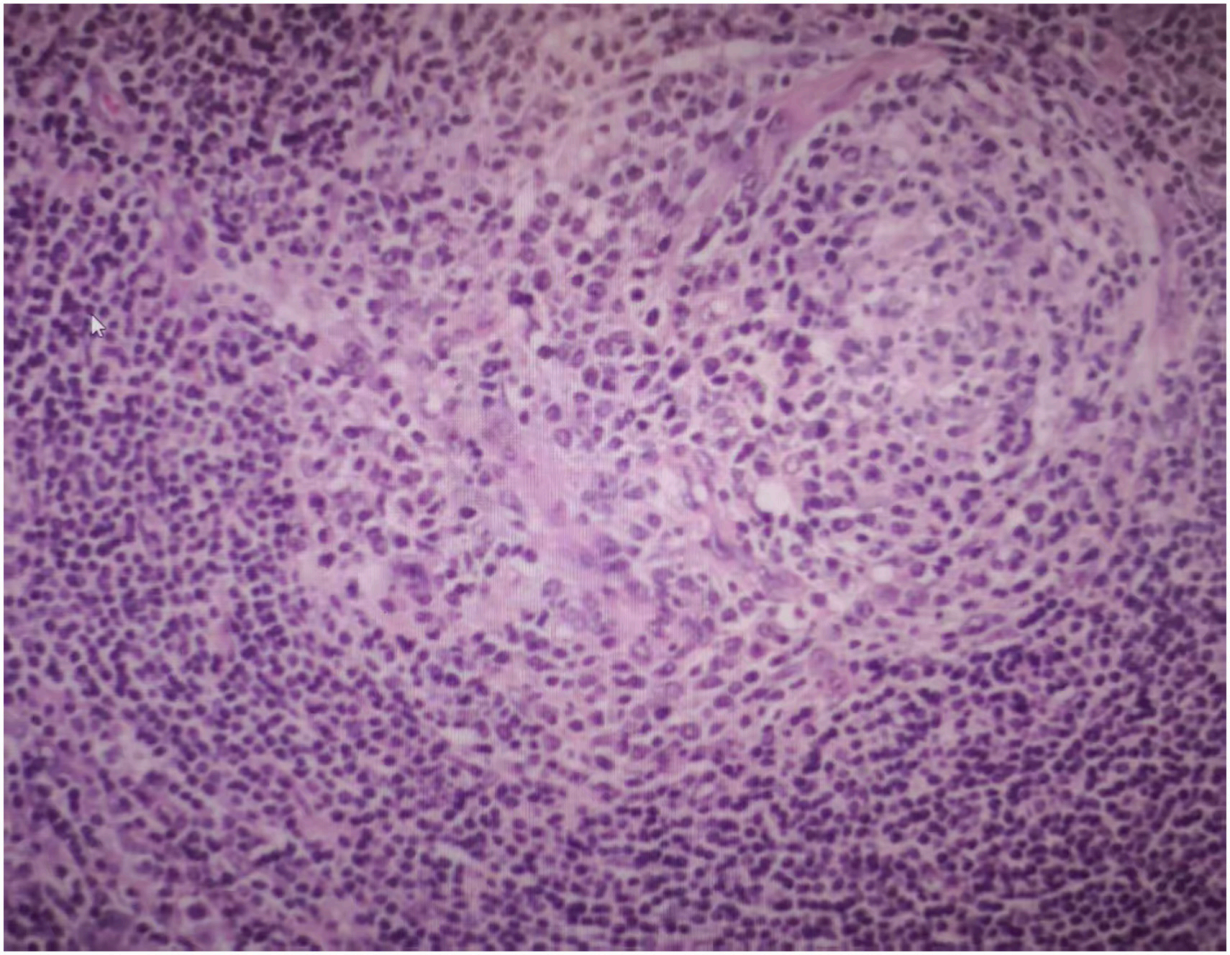
Postoperative pathological examination showed the mass is UCD, hyaline-vascular type.

Three days after being admitted to hospital, the patient subsequently underwent surgery to completely resect the retroperitoneal mass under general anesthesia. A large mass, approximately 10 cm × 6 cm in size, was detected in the left retroperitoneum. It was well-defined and showed no infiltration into surrounding tissues, allowing for complete excision. Postoperative pathological examination confirmed the diagnosis of UCD, hyaline-vascular type (Figure 3). Immunohistochemical staining results were as follows:CD20 (lymphoid follicle cells +), CD79a (lymphoid follicle cells +), CD3 (interfollicular area cells +), CD5 (interfollicular area cells +), CD21 (follicular dendritic network +), Bcl-2 (follicular dendritic network and surrounding lymphocytes +), CD10 (focal +), CD138 (focal +), Cyclin D1 (scattered +), CD30 (individual cells +), Ki-67 (+).

The patient recovered well postoperatively and received oral glucocorticoid therapy, with the dosage gradually tapered until discontinuation. At the 1-year follow-up, the oral erosions and hand erosions had healed. After 2 years of follow-up, there was no recurrence of the oral erosions or hand erosions, and an abdominal CT scan showed no evidence of recurrence.

## Discussion and literature review

Unicentric Castleman disease is a rare disease characterized by single enlarged lymph node or several enlarged lymph nodes within a single lymph node station (20). According to a systematic review of 404 patients, UCD is more likely to be detected in the mediastinum (29%), neck (23%), abdomen (21%), and retroperitoneum (17%), but it can also be detected in any lymph node throughout the body (7). UCD is usually asymptomatic and mostly detected due to compression of adjacent structures (e.g., airways, blood vessels, nerves, ureters) or incidentally through imaging examinations (20–22). CT scan of the of the neck, chest, abdomen, and pelvis can clearly show the enlarged lymph nodes. CT–positron emission tomography scanning can also help locate the lymph nodes by monitoring the metabolic activity (20, 23, 24). Laboratory tests are usually normal, but anemia, hypergammaglobulinemia and elevated sedimentation rate may be present (22).

Biopsy is the gold standard for the diagnosis of CD (20). An excisional biopsy instead of core or fine-needle aspirate is needed to get the sample of the enlarged lymph node (3, 20). Based on histopathological features, CD is primarily classified into the hyaline-vascular (HV) type, plasma cell (PC) type, and mixed type (20). UCD is predominantly characterized by the HV histopathologic type, which is observed in 70%–90% of UCD patients. The plasma cell or mixed type is identified in approximately 10%–30% of UCD cases (22). Those patients can present with symptoms similar to those of MCD, such as sweats, fever, anorexia, or weight loss (20, 25).

In the present case, the patient reported no abdominal discomfort. Despite persistent growth, the mass did not compress any adjacent organs or tissues to become clinically apparent. Moreover, UCD patients, particularly those with the hyaline-vascular type, are frequently asymptomatic, making the mass difficult to detect (20, 22, 26). In preoperative examinations, the biochemical profile, full blood count, serum antibodies, and coagulation profile were all normal; however, the squamous cell carcinoma-associated antigen level was elevated, while in another case repot carcinoembryonic antigen is elevated (27). These largely normal findings initially led to a misdiagnosis of isolated mucosal lesions, resulting in a delay of several months before the correct diagnosis was established. Approximately sixty percent of patients present with oral manifestations prior to the diagnosis of an underlying neoplasm, only a small percentage of patients had oral manifestations concurrently with the diagnosis of neoplasm (10). When associated with PNP, UCD patients can present with severe stomatitis, frequent painful ulcers on the tongue and erosions on the lips that extend to the vermillion border of the lips (14). These lesions are refractory to prolonged symptomatic therapy, which is a hallmark of PNP (10, 11). Therefore, for such patients, it is crucial to consider a possible diagnosis of paraneoplastic pemphigus. If PNP is suspected, CT imaging serves as a valuable tool for identifying potentially enlarged lymph nodes or tumors to achieve an early diagnosis (8, 9).

Greater clinical attention is warranted for UCD patients with PNP (3). Unlike those with isolated UCD, these patients face an increased risk of respiratory failure and pulmonary infection, which are the leading causes of mortality (12, 17). Management should address not only the symptomatic control of PNP-associated mucosal lesions but also perioperative administration of high-dose intravenous immunoglobulin to prevent pulmonary complications, particularly BO (28). Our patient received both oral glucocorticoid therapy and intravenous immunoglobulin perioperatively. Additionally, a chest CT scan was performed to screen for any pulmonary involvement.

Complete surgical resection is the primary recommended therapy for patients with UCD and PNP (3, 20). Surgery serves to remove the mass, alleviate potential symptoms caused by compression of adjacent tissues, improve oral lesions, and halt the progression of PNP (9, 19). Preoperative evaluation should focus on assessing the feasibility of complete resection and weighing the surgical risks against benefits (20). If complete resection is not feasible, partial resection may be considered. Following partial resection, rituximab in combination with corticosteroids can help reduce the size of the residual mass, potentially meeting the criteria for a subsequent complete resection (3). Patients may benefit from a second surgery for complete resection if reevaluation after adjuvant drug therapy deems them suitable. The vast majority of UCD patients are cured after complete resection, with recurrence being rare (29–31). According to global multicenter reports, the 5-year survival rate for UCD patients after complete resection exceeds 90% (5–7). A 20-year French study of 273 CD patients reported a 2-year survival rate of 98.1% for UCD patients (26). Similarly, a retrospective study from China reported 1- and 5-year survival rates of 98.5% and 97.1%, respectively, for UCD patients (5). Postoperatively, treatment with oral systemic corticosteroids, topical treatment of lesions and annual follow-up is recommended (3, 12). The follow-up includes CT scan, complete blood count, lactate dehydrogenase, as well as assessments of liver and renal function, electrolytes, albumin, CRP, and quantitative immunoglobulins (3).

For patients with unresectable UCD after other therapies have been considered or attempted, radiation therapy may be an option, though it is often unsuitable for younger patients (32). A recent publication from the Mayo Clinic suggested that cryoablation could be a potential treatment for unresectable UCD (33). For patients assessed preoperatively as having a high risk of hemorrhage, angiographic embolization may be performed (3).

Overall, patients with PNP accompanied by CD have a relatively favorable prognosis (9, 18). Nevertheless, some patients may still experience pulmonary involvement, which can lead to respiratory failure or even death (19). The prognosis of PNP is generally poor. When associated with malignancy, the mortality rate approaches 90% (28). With early and complete surgical resection, such patients can achieve long-term remission, as evidenced by multi-year follow-up (19). In our case, the patient underwent complete resection, and two-year follow-up has shown a favorable prognosis without recurrence.

## Conclusion

Unicentric Castleman disease associated with PNP represents a complicated clinical scenario, in which early diagnosis is crucial to intervene before the onset of BO and respiratory symptoms. Clinicians should consider the possible diagnosis of PNP in patients whose oral lesions show no improvement after symptomatic treatment. Complete surgical resection remains the first-line recommended therapy for these patients. Overall, with early recognition and timely operative management, the prognosis is generally favorable.

## Data Availability

The original contributions presented in this study are included in this article/supplementary material, further inquiries can be directed to the corresponding author.
